# The Implications of Habitat Management on the Population Viability of the Endangered Ohlone Tiger Beetle (*Cicindela ohlone*) Metapopulation

**DOI:** 10.1371/journal.pone.0071005

**Published:** 2013-08-12

**Authors:** Tara M. Cornelisse, Michelle K. Bennett, Deborah K. Letourneau

**Affiliations:** Environmental Studies Department, University of California Santa Cruz, Santa Cruz, California, United States of America; The Australian National University, Australia

## Abstract

Despite their role in providing ecosystem services, insects remain overlooked in conservation planning, and insect management approaches often lack a rigorous scientific basis. The endangered Ohlone tiger beetle (*Cicindela ohlone*) occurs in a 24-km^2^ area in Santa Cruz County, California. The once larger metapopulation now consists of subpopulations inhabiting five patches of coastal prairie where it depends on bare ground for mating, foraging, and oviposition. Human activities have eliminated natural disturbances and spread invasive grasses, reducing *C. ohlone*'s bare-ground habitat. Management actions to restore critical beetle habitat consist of cattle and horse grazing, maintaining slow bicycle speeds on occupied public trails, and artificial creation of bare-ground plots. Recreational biking trails help maintain bare ground, but can cause beetle mortality if left unregulated. We tracked *C. ohlone* survivorship and estimated fecundity for three years. We then constructed a stage-structured population projection matrix model to estimate population viability among the five patches, and to evaluate the success of management interventions. We demonstrate that habitat creation, regulation of bicycle speed, and migration between patches increase *C. ohlone* survival and population viability. Our results can be directly applied to management actions for conservation outcomes that will reduce species extinction risk and promote recolonization of extirpated patches.

## Introduction

Although the ultimate causes of species endangerment are most commonly habitat loss and invasive species introductions [1], proximate causes are reduced viability of small populations via genetic degeneration and demographic and environmental stochasticity [2]–[4]. Thus, while habitat protection is vital to species conservation, management is often needed to ensure viability of populations within protected habitat and across landscapes [5]–[8]. It is particularly important to understand the effect of management actions on tangible population viability goals of endangered species to ensure efficient and effective use of resources to prevent species extinctions [4], [9], [10].

Despite their significance in ecosystem functions, insects are frequently overlooked in conservation actions, and endangered insect recovery plans often lack quantitative population goals to ensure long-term viability [11], [12]. More than 20 species of tiger beetles (Coleoptera: Cicindelinae) have been listed as threatened, endangered, or extinct worldwide and many more as US federal species of concern [13]. Pearson et al. [14] estimate that at least 33 (15%) of the 223 named species and subspecies of tiger beetles in the US and Canada may be declining at a rate that justifies their listing as threatened or endangered. Tiger beetles are associated with distinct disturbance-dependent bare-ground habitats needed to forage, find mates, and oviposit; thus, while they are sensitive to habitat degradation, they are increasingly dependent on anthropogenic disturbance [13], [15].

The endangered Ohlone tiger beetle metapopulation (*Cicindela ohlone* Freitag and Kavanaugh) is endemic to the coastal prairies of Santa Cruz County, California. The remaining *C. ohlone* populations are present in habitat patches of a once more extensive metapopulation that consisted of 10–15 patches in the last 25 years [16]. Adult *C. ohlone* are generalist predators that stalk and chase down prey in open areas using visual cues, and larvae are sit-and-wait predators that construct a cylindrical burrow flush with the soil surface from which they lunge to capture passing arthropods; thus, both require bare ground for capturing prey [15]. The coastal prairie habitat evolved with disturbances such as large ungulate grazing and fires that created favorable conditions for the beetle's bare-ground habitat [17], [18]; however, human activities have eliminated natural disturbances and spread invasive grasses, which form dense, extensive stands, reducing the incidence of bare ground [19], [20].

Management of livestock grazing, recreation and artificial habitat creation currently maintain bare-ground in the remaining *C. ohlone* habitat patches. Creation of bare-ground plots by scraping the ground surface free of vegetation successfully augments egg-laying habitat for *C. ohlone* [21]. However, tiger beetle larval habitat augmentation within areas already limited by quality habitat could create a potential for negative density-dependence, with increased larval density leading to increased competition and reduce larval survivorship [22]. Recreational (i.e. hiking and cycling) trails also create bare ground, but high-speed cycling can disrupt the mating and foraging behaviors of *C. ohlone* adults (unpublished data). Thus, while systematic management of bare ground creation and recreation maintains *C. ohlone* habitat, it is unknown how these actions affect *C. ohlone* population viability.

The metapopulation dynamics of *C. ohlone* may be compromised because of habitat destruction and decline of habitat quality resulting in a few remnant populations; but because there have been two population turnover events in recent years, we have reason to believe *C. ohlone* has the capability of recolonization provided suitable habitat is available and managed in extirpated patches. However, recolonization of extirpated patches depends on asynchrony of *C. ohlone* population dynamics and their ability to migrate between populations [23], both of which are unknown.

We use population viability analysis (PVA) to model the effects of conservation management and metapopulation dynamics on *C. ohlone* viability. We predicted that artificial bare ground creation and managed recreation would augment the population growth rate of all *C. ohlone* populations when accounting for density-dependent effects, likely a significant factor in determining larval survival. We also predicted that *C. ohlone* vital rate dynamics would be asynchronous among populations and that any migration would reduce *C. ohlone* extinction risk. To test our predictions, we used PVA to model the growth rates of all *C. ohlone* populations and the associated vital rate sensitivities. We also determined how management strategies and metapopulation dynamics affected each rate effort to plan for the recovery of this endangered species.

## Methods

### Study sites

We conducted this study from January 2010 to August 2012 within the five remaining populations of *C. ohlone*, located in different coastal terrace prairie sites within a 24 km^2^ area in Santa Cruz County, California: Lower Marshall (LM, 1.5 ha, 37.02°N 122.07°W) and Wilder Ranch (WR, 3.5 ha, 37.01°N 122.09°W), 1.3 km apart in the center of the range; Moore Creek (MC, 9.6 ha, 36.97°N 122.07°W) and University of California Campus (UC, 7.8 ha 36.98°N 122.07°W), 0.75 km apart in the south of the range; and Glenwood (GW, 2.9 ha, 37.07°N 121.99°W), 10 km north of the other sites. Santa Cruz County has a Mediterranean climate that receives an average of 77 (58–120) cm of rain, 95% of which falls from October to April. During this study, the annual precipitation was 99.8 cm, 72.6 cm, and 74.8 cm from 2010 to 2012, respectively. While the California coastal prairies can experience a rare fire or extreme drought, to our knowledge, the weather fluctuations experienced during this study represent the average conditions. This work was completed under USFWS permit #TE-39184A-0.

### *Cicindela ohlone* lifecycle

Adult *C. ohlone* emerge from oviposition burrows, oviposit, and are active from late January to May. From February through early April, females deposit eggs singularly in the soil and the larvae develop at the site of oviposition. The first instars hatch in April through May, remaining in the first instar stage for four to six weeks [15], [24], then progress to the second instar in May through June. Development to the third instar almost always occurs during the same summer, in July, after which the third instar plugs its burrow and pupates in late September through January, completing a one-year cycle. In addition, a few individuals have been observed to delay pupation until the following spring when the third instars unplug their burrows after winter inactivity [24].

### Data collection

**Figure 1:**
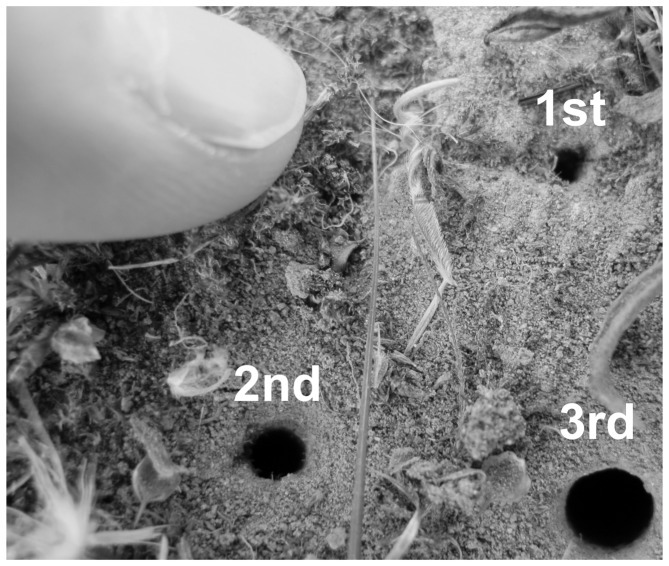
Burrows of the three Ohlone tiger beetle instars with an index finger for scale.

We surveyed adults once or twice per week late January to early June 2010 to 2012. We did not include UC in 2010 because we thought the beetles were extirpated from that site at the time. We estimated the number of *C. ohlone* adults and surveyed for larval burrows using a visual index count [25]. First instar larval burrows were surveyed in March to late April 2010 to 2012, identified by burrow diameter (Fig. 1) and assumed to be active if it was clearly delineated with a clean entrance, a sign of recent larval activity [15]. We overlaid the burrow(s) with a 0.25 m^2^ gridded quadrat, marked the corners with a 3-cm wide metal washer and 5-cm long nail and the locations with a GPS to avoid disturbing larvae. We mapped all burrows in the quadrat on a gridded datasheet resembling the quadrat. In 2012, we identified and marked oviposition burrows in early March in the same manner as first instars. In 2010 and 2011 we marked 20 quadrats at each site and in 2012 we marked nine quadrats of oviposition burrows and 18 quadrats of first instar burrows, for a total of 27 quadrats at each site, which was dictated by the number of burrows found during surveys.

To determine if eggs survived to first instar, first to second, and second to third, or remained in the same stage, we revisited each site in late April and May, June, and July, respectively, sufficient time for all stage transitions to occur [15]. Quadrat markers were located via GPS and then either visually or using a metal detector. We lined up the gridded quadrat to the metal markers and considered the transitions to have occurred based on the increase in burrow entrance diameter, which the larva enlarges after molting to the next instar (Fig. 1). The following year, we revisited the locations of the previous year's third instar burrow once per week from late January though mid-March to check marked burrows for third instar larva survival and transition to adults by the presence of an irregular exit hole ≥6 mm.

### Parameter estimates

**Figure 2:**
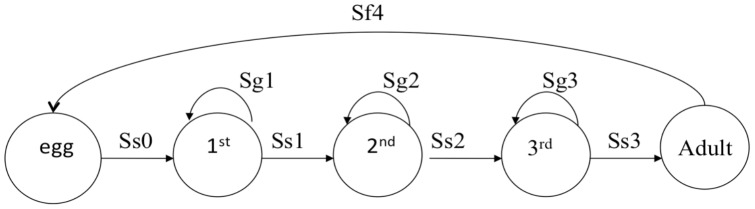
Ohlone tiger beetle stage class population model. The parameters presented are those we measured: survival of egg to 1^st^ instar (Ss0), growth of 1^st^ instar (Sg1), survival of 1^st^ to 2^nd^ instar (Ss1), growth of 2^nd^ instar (Sg2), survival of 2^nd^ to 3^rd^ instar (Ss2), growth of 3^rd^ instar (Sg3), survival of 3^rd^ instar to adult (Ss3), and fertility of adults (Sf4).

We counted the total number of adults surveyed as females in the projection matrix model because visual index counts generally underestimate tiger beetle adults by ∼50% [25] and sex ratios are not significantly different from 1∶1 [16], [25]–[27]. We assigned fertility rate (Sf4) as 40 because it is the best estimate of the mean total number of eggs a female *C. ohlone* oviposits in her lifetime [24]. We calculated larval growth rate within a stage (Sg*i* , *i*  =  instar stage 1, 2, or 3) as the proportion of individuals in the same stage at the next census. Transition rates (Ss*i* , *i*  =  the stage that transitioned to instar stage 1, 2, 3, or adult) were calculated as the proportion of individuals that transitioned from one stage to the subsequent stage in the next census [9]. Adult survival rates were included as zero because all adults senesce during the activity year. To estimate fecundity (F), we averaged three different estimates: (1) assuming a breeding pulse and mid-breeding census of eggs by multiplying the fertility rate by the square root of egg survivorship (Ss0); (2) assuming a constant breeding flow and mid-breeding census by multiplying the fertility rate by both the square root of (Ss0) and the square root of an estimate of adult survivorship; and (3) assuming a post-breeding census by multiplying the fertility rate by Ss0 (See [9], Ch. 6 for explanation of assumptions). We constructed a stage-class population model for *C. ohlone* (Fig. 2) and used the model parameters to construct a stage-class matrix model:
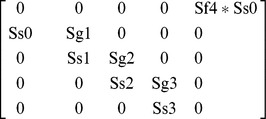



### Density dependence

To test for density dependent effects on larval survival, we used a random number generator to select an individual in each of the marked quadrats. We recorded survivorship from first to second and from second to third instar as survived (1) or dead (0) for each selected individual as well as the number of larval burrows in each quadrat surrounding the selected individual. Individuals still in the second instar stage during the last field visit were marked as survived (1). We tested the effect of larval density on the survival of selected individuals among sites using logistic regressions. We assumed prey was the most likely limiting factor involved in any inter-larval competition [28], [29]. Larval density was log-transformed to fulfill assumptions of normality. Logistic regressions were carried out using SPSS v. 19.0.0 (SPSS, Inc., IBM).

### Matrix modeling

All matrix analyses were done using Matlab Student Version 7.12 and methods described in Morris and Doak (2002) [9]. To account for demographic and environmental stochasticity, we constructed one matrix per study year and conducted the multiple matrices approach to estimate population growth rate [30]. Stochastic log growth rate, log λ_s_, was determined both via simulation using the program stoc_log_lam and using Tuljapurkar's approximation (τ^2^), which accounts for the covariance and variability of matrix elements among years. [9], [31]. We assumed all matrices had equal probability of occurring and simulated 50,000 iterations. We used simext.m (Box 7.5 [9]), to evaluate the fraction of simulated populations that reach the quasi-extinction threshold after a designated time t_max_ set to 25 and 50 years and the quasi-extinction threshold to 25, 10, and one individual(s) and weighted all matrices equally.

### Sensitivity analysis

We conducted a stochastic sensitivity and elasticity analysis for each population by simulating multiple matrices using low, average, and high estimates of each vital rate using limitsens.m (Box 9.2 [9]). Maximum likelihood estimates of Ss1, Ss2, and Ss3 were calculated using Kendall.m [32] (Box 8.2 [9]) and we used the resulting confidence interval values as our high and low estimates of the survivorship vital rates in limitsens.m. Vital rates associated with the three measures of fecundity were used for fertility estimates; 40 for average, 60 for the high (highest estimated by [24]), and lowest fecundity value for the low estimate.

### Sensitivity to management effects

To test the effect of creation of bare-ground and mandated slower cycling in *C. ohlone* habitats on population growth rates, we explicitly included a management scalar, *h*, in a deterministic matrix model using the program vitalsens.m (Box 9.1 [9]), in which we also calculate sensitivities and elasticities of *h* to each population growth rate. We averaged vital rates for all years and used 40 for the fertility value in the matrix.

Because female *C. ohlone* will lay up to 60 eggs in captivity [24], we assumed females would lay 60 eggs with increased bare ground. This is justified because creating bare ground in *C. ohlone* habitat will likely increase the number of eggs laid by females, as up to eight times more larval burrows were found in scraped ground compared with vegetation-covered controls [21]. Females will also obtain more food with more bare ground (e.g. hunting ground), increasing their fecundity and egg survivorship [15], [29]. Furthermore, larvae that develop in bare ground experience increased prey availability and, in turn, reduced development time from 160 to 110 days, a 30% reduction [13], [15], [22], [24], [29]. Thus, we included bare ground creation as a management strategy that increases the survivorship and growth of eggs, 1^st^, 2^nd^, and 3^rd^ instar by 30%, *h* = 1.3, and the number of eggs by 1.5 times (1.15*h). The resulting matrix is:
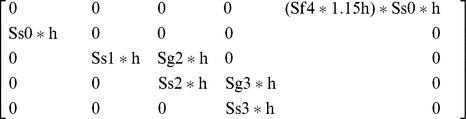



We also modeled a hypothetical strategy that required all cyclists to slow down to speeds of 8–12 kph in *C. ohlone* habitats that allow bicycles: UC, LM, and WR. Reducing bicycle speed to 8–12 kph has the potential to reduce recreational disruptions to adult mating and foraging behavior (unpublished data), increasing adult survivorship and the number of eggs laid because of both increased fertility and increased reproductive period [15], [29]. We incorporated the management strategy of reducing the bicycle speed allowed in *C. ohlone* habitat by including a scalar, *h*, to the matrix vital rates that increased the number of eggs laid to 80. This is justified in that tiger beetles are able to lay up to 200 eggs per female lifetime if a female lives for 30 days, an estimate for the average life span for adult tiger beetles [15]. We also increased the fecundity, or survivorship of eggs, by 30%, as justified above. The resulting matrix is:
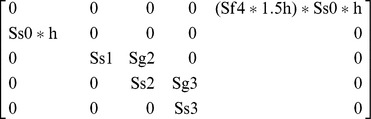



### Metapopulation dynamics

We calculated vital rate correlation coefficients among sites from 2010–2012 using Pearson's correlation to check for asynchrony. To determine the overall metapopulation growth rate as well as the quasi-extinction risk, we created a metapopulation matrix composed of the individual population vital rates and used DemoMetaSim.m (Box 11.5 [9]). We capped the egg and larval stages at 100 individuals and adults at 300, high estimates of observed numbers, and the quasi-extinction thresholds to five, 10, and 20 individuals in each stage, and maximum time to 100 years for 500 runs. We ran the program first assuming no migration and including all sites and then by excluding sites one by one to test the extirpation or complete isolation of each site.

We also simulated quasi-extinction probability for LM and WR alone, as they are clumped in space. We simulated population growth and quasi-extinction risk for WR and LM assuming no migration, assuming one out of 50 adult females migrate between the two sites, or m = 1/50 = 0.02; one out of 25, or m = 1/25 = 1.04; and one out of 10, or 10% m = 1.10; thus, each adult vital rate (Sf4) was multiplied by 1.02, 1.04, and 1.10, respectively.

## Results

The number of adults, marked burrows, and vital rates varied between sites and years (Table 1). Surviving first instar larvae never remained in the 1^st^ instar stage between two successive censuses, thus we did not include a measure of Sg1 in the models. Egg survivorship estimates were similar between all populations except WR where we found the lowest estimate (Table 1), resulting in lower fecundity estimates. By averaging the three fecundity measurements described in the methods, we obtained the following fecundity (F) estimates for each population: GW 22.9 (±6.51); LM 14.8 (±5.81); WR 6.49 (±3.08); MC 19.7 (±4.17); UC 26.3 (±3.49).

**Table 1:** Number of Ohlone tiger beetle eggs and first instars marked (all quadrats combined), number of adults counted, and growth and survival parameters for each stage and site (see text for parameter explanation and calculation method).

	Glenwood (GW)			Lower Marshall (LM)			Wilder Ranch (WR)			Moore Creek (MC)			Campus (UC)	
Year	2010	2011	2012	2010	2011	2012	2010	2011	2012	2010	2011	2012	2011	2012
**Eggs**	na	na	58	na	na	31	na	na	36	na	na	27	na	28
**1^st^instars**	69	99	159	48	117	67	110	66	59	125	79	72	80	88
**Adults**	41	39	226	59	51	68	124	25	86	100	64	428	67	166
**Ss0**	na	na	0.59	na	na	0.32	na	na	0.06	na	na	0.41	na	0.54
**Ss1**	0.45	0.69	0.94	0.79	0.51	0.67	0.29	0.38	0.71	0.22	0.54	0.60	0.51	0.74
**Sg2**	0	0	0	0.05	0.03	0.27	0.03	0	0.05	0	0	0.02	0	0.2
**Ss2**	0.74	0.85	0.80	0.42	0.53	0.51	0.31	0.52	0.57	0.54	0.77	0.70	0.68	0.45
**Sg3**	0.74	0.21	0.20	0.75	0.69	0.69	0.3	0	0	0	0	0	0.14	0.14
**Ss3**	0.26	0.64	0.75	0.13	0.25	0.25	0.7	0.85	0.85	1.0	0.88	0.88	0.82	0.82

na indicates not marked that year.

### Density dependence

The number of larvae in a quadrat ranged from 1–13, with an average of 3.3 (±2.4). There was no effect of larval density (number of larval burrows in a quadrat), site, or site × larval density interactions on survivorship of first instars to second (R^2^ = 0.029, p = 0.469), nor second instars to third (R^2^ = 0.015, p = 0.692). Thus, we found no evidence of density-dependent larval survival.

### Matrix modeling

The simulated growth rates (λ_s_) with 95% confidence intervals were: GW 1.41(1.405–1.415); LM 1.03 (1.028–1.032); WR 0.598 (0.596–0.601); MC 1.164 (1.153–1.174); and UC 1.163 (1.153–1.174). The growth rates calculated by Tuljapurkar's approximation were within the 95% confidence intervals of λ_s_. For every population except WR, the stochastic quasi-extinction rate was zero in all time frames modeled. In all time frames and quasi-extinction thresholds, the extinction probability of the population at WR was 1.00, or definite extinction, by 21 years. The quasi-extinction probability or reaching 25 and 10 individuals was 1.00 by eight and 14 years, respectively.

### Sensitivity analysis

**Figure 3:**
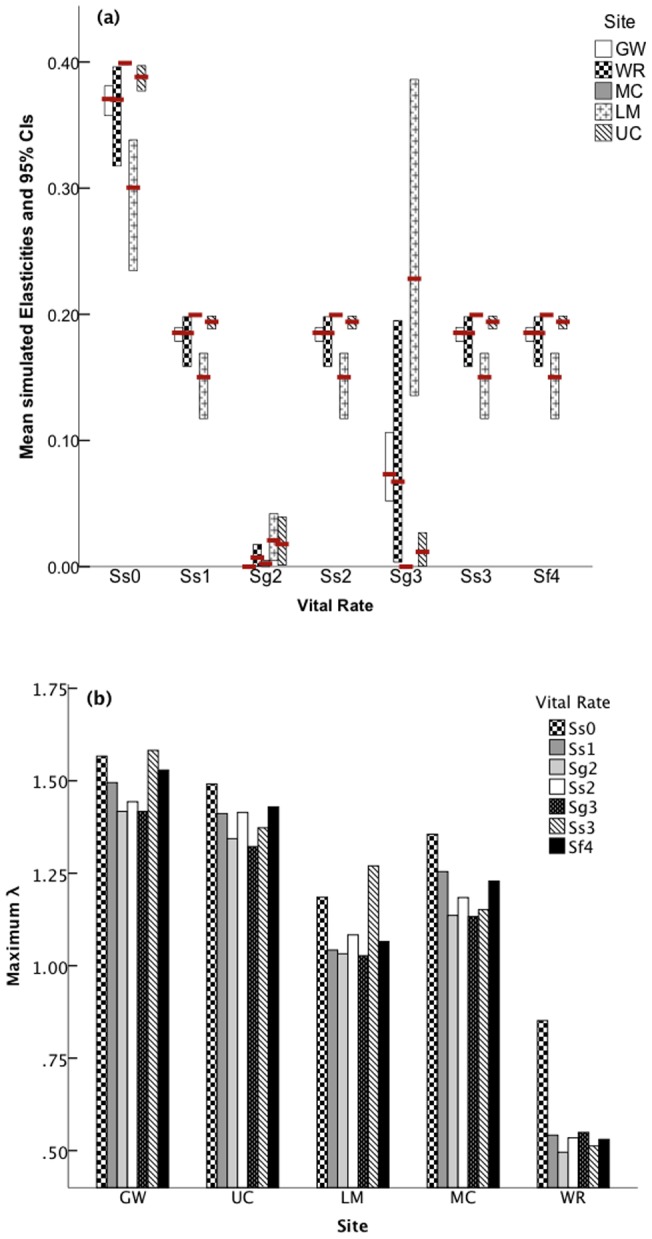
Vital rate elasticities as they relate to population growth rates (λ) (a) and the maximum possible population growth rate (λ) obtained when maximizing each vital rate (b), for each site. Red bars represent mean elasticities. See text for vital rate meanings.

The elasticities of Ss1, Ss2, Ss3, and Sf4 were equal with relatively small confidence intervals, whereas Sg2 elasticities were very small with narrow confidence intervals (Fig. 3a). Egg survivorship (Ss0) and 3^rd^ instar growth (Sg3) elasticities varied greatly among populations and uncertainties of those parameters were high in both LM and WR, as indicated by the wide confidence intervals (Fig. 3a).

For UC, MC, and WR, maximizing the survivorship of eggs has the greatest potential to maximize population growth rate (Fig. 3b) with corresponding r^2^ values (a measure of influence on the population growth, λ) of: 0.4437, 0.3941, and 0.4503, respectively. The population growth rate of WR remains below λ = 1 despite maximizing egg survivorship (Fig. 3b). For GW and LM, maximizing both egg survivorship (r^2^ = 0.5917 and 0.6941) and survival of 3^rd^ instars (r^2^ = 0.0224 and 0.0918) maximized population growth rate (Fig. 3b).

### Sensitivity to management effects

**Figure 4:**
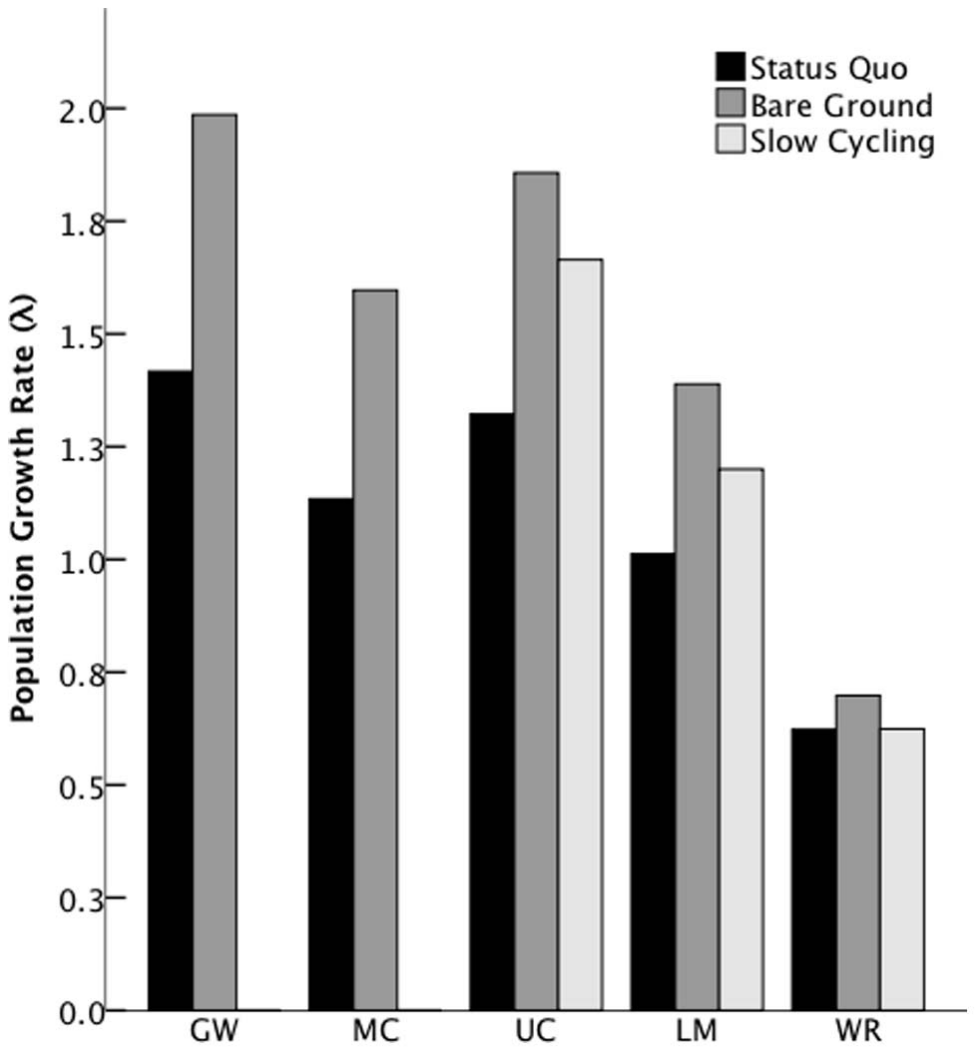
Population growth rates for all populations with current management (Status Quo (λ_1_)), with increased bare ground, and with slower cycling where recreation is permitted.

The simulated management strategies had positive effects on the growth rates of all populations, yet varied in their magnitude among the populations (Fig. 4). Sensitivities (and elasticities) for bare ground *h* were: GW 1.81 (1.19); MC 1.47 (1.20); UC 1.70 (1.19); LM 1.22 (1.14); WR 0.639 (1.19). Sensitivities (and elasticities) for slow cycling *h* were: UC 0.745 (0.582); LM 0.423 (0.458); WR 0.275 (0.572). Thus, while reducing bicycle speed resulted in a smaller increase in growth rates compared to increasing bare ground, both only marginally increased the WR growth rate (Fig. 4).

### Metapopulation dynamics

The Pearson correlation analysis revealed that while vital rates were largely correlated among sites, Ss1, Ss2, and Ss3 were asynchronous between LM and all other sites, WR and UC, MC and GW, and MC and LM, respectively.

The simulated metapopulation growth rate ranged from 1.2935 to 1.6282 and the quasi-extinction probability was zero for all simulations containing the GW, MC, and UC populations. Since all combinations of sites that included GW, UC, and MC had a positive population growth rate and a zero chance of quasi-extinction at any threshold with no migration we did not simulate migration including these sites because migration only acts to decrease the risk of extinction, with our evidence of no negative density dependence.

**Figure 5:**
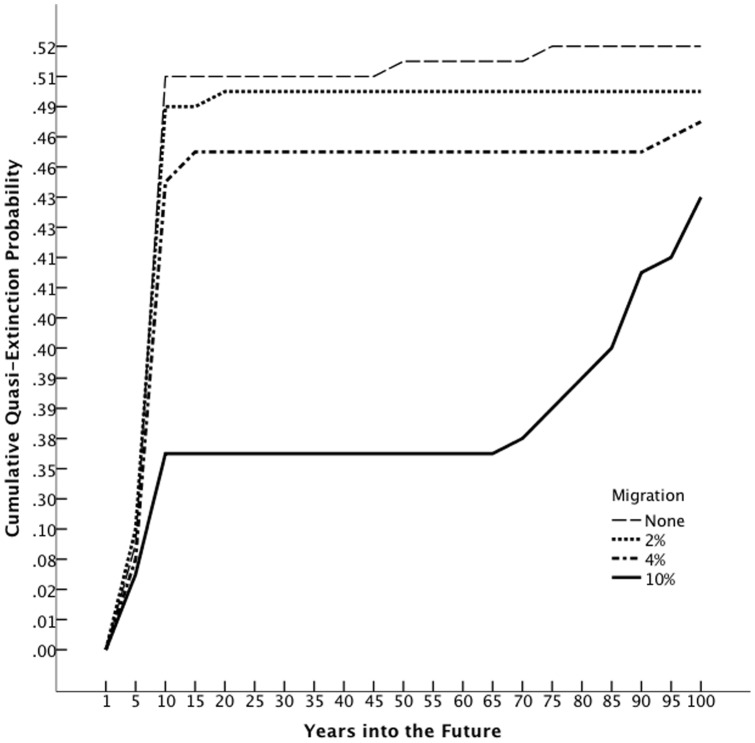
Cumulative quasi-extinction probability (set at ten individuals per stage class) for WR and LM populations combined with different migration scenarios.

The maximum population growth rate and probability of quasi-extinction for WR and LM in 100 years, assuming 10 individuals in each stage and no migration, was 0.9396 and 0.5160, respectively (Fig. 5). Assuming 2% migration, or one per 50 adult females migrate between the two sites, population growth was 1.0131 with quasi-extinction probability reduced to 0.4880 in 100 years; for 4% migration, the growth rate was 1.0115 and quasi-extinction probability 0.4640 in 100 years; for 10% migration, the growth rate was 1.0133 and quasi-extinction probability 0.4320 in 100 years (Fig. 5). Thus, migration increased the population growth rate for WR and LM to λ>1and reduced the quasi-extinction probability by 5–10%.

## Discussion

The endangered Ohlone tiger beetle represents the fragmented status of many threatened species for which coordinated, scientifically based and data driven management is desperately needed. By understanding population growth between and among populations as well as the effect of management strategies, we were able to evaluate the consequences of conservation actions on the recovery of *C. ohlone*.

Environmental and demographic stochasticity did not appear to be important factors of *C. ohlone* population growth, as matrix elements were not highly variable among years, Tuljakurpur's approximation fell within the narrow confidence intervals of the stochastic population growth, and we found no evidence of a density-dependent effect on larval survival. Despite these findings, environmental stochasticity should not be ignored as we only have three years of data, insufficient to predict insect population fluctuations [33]. In addition, the impact of environmental stochasticity on variable population growth will increase as global warming continues to alter climate patterns around the world [4].

The stochastic projection matrices revealed some large differences in growth rates among *C. ohlone* populations. GW had the highest growth rate, yet since it is the most physically isolated site, metapopulation theory would predict it to be at high risk of extirpation [23]. While several studies show that metapopulation theory is useful in explaining some patterns of extinction, many show that local scale, within patch, habitat characteristics are important for the conservation of insect species [34]–[40]. GW is grazed by horses year round and has a high percentage of bare ground and low standing vegetation (unpublished data). GW is also managed by the Land Trust of Santa Cruz County, which employs biological consultants to improve the land specifically for *C. ohlone* viability. Thus, GW is an example of how extinction risk of isolated patches can be reduced with increased habitat quality and management; yet, long-term persistence of GW as part of the metapopulation will require understanding of *C. ohlone* dispersal ability and rates between patches.

WR was the only population that had a negative population growth and high extinction risk across all projected models. While it seems that the reason for this was the low egg survivorship, all WR vital rates were generally lower than for other populations. Indeed, if we substitute the egg survivorship and mean fecundity values from LM (which is mostly likely a high estimate for true WR values) into the WR population projection matrix, the growth rate remains below 1.0 at 0.97. In contrast to GW, WR represented how a more connected habitat patch can become non-viable as habitat quality declines, suggesting that increased habitat management in WR could increase *C. ohlone* viability.

The sensitivity analyses revealed that, for all populations, the population growth rate was most sensitive to egg survivorship. The wide confidence intervals around the egg survivorship estimates for LM and WR indicated a greater uncertainty in those values and while we recognize the limited predictive power of a single estimate of egg survivorship, our estimates fall well within those published for tiger beetles [41]. The LM population growth rate was also highly sensitive to the growth of third instar larvae. This pattern is important for viability in that larvae will prolong their pupation without sufficient food, increasing their risk of mortality [15], [25]. As the LM population growth rate was near 1.0, management that augments food availability, such as increased bare ground, may become important for population viability.

Increasing bare ground and requiring cyclists to slow down in *C. ohlone* habitat created large, positive changes in all populations. We based our assumed increase of 1.5 times the number of eggs laid on our previous study [21], which follows that as little as 9-m^2^ of bare ground per site would be beneficial. Similarly but less so, management that requires a reduction in bicycle speed increased population growth rates in the three sites that allow bicycle recreation. We assumed that this management action only affected fecundity because recreation mainly affects the mobile adult stage. Our elasticity results showed that reducing bicycle speed to 8–12 kph increased population growth by 42–58%.

We found that any amount of migration among *all* sites ensured that the *C. ohlone* metapopulation would not go extinct within the next 100 years, assuming current management remains in effect. Despite the fact that vital rates were generally asynchronous among sites regardless of the distance separating them, *C. ohlone* dispersal dynamics are unknown and because remaining patches are fragmented, we are unsure if *C. ohlone* has sufficient colonization ability. Thus, future studies should focus on understanding *C. ohlone* dispersal ability and rates in order to quantify recolonization potential or the need for translocation.

When we removed the populations with high growth rates, GW, UC, and MC, equivalent to no migration between these populations and the other two, WR and LM had a 35% probability of quasi-extinction in the next 10 years, even with a high degree of migration. WR and LM are in a habitat cluster that has lost two populations in recent years [16], [24]; this increased isolation could be contributing to the non-viable status of WR and low growth rate in LM via inbreeding depression. Fortunately, the coastal prairie habitat between these two sites is protected but management efforts focused on augmenting habitat quality in extirpated patches would be a necessary first step in promoting recolonization and maintaining *C. ohlone* viability in this area.

*Cicindela ohlone* declined across the landscape due to habitat destruction prior to its listing as an endangered species; however, as is the case with many threatened species, protection of occupied habitat alone is not enough to prevent its extinction [6], [8]. At the site level, management actions that maintain bare ground and reduce incidental mortality of *C. ohlone* must be in place to maintain stable populations [13], [21], whereas at a landscape level both recently extirpated sites and potential coast prairie habitat should be managed to maintain suitable *C. ohlone* habitat for future colonizations. We are currently analyzing the potential for unoccupied sites to contribute to *C. ohlone* habitat and range expansion. The results of this study clearly illustrate that *C. ohlone* has four viable populations that, with habitat management, could recolonize extirpated sites and avoid species extinction.
